# Cloning and Functional Characterisation of the Duplicated RDL Subunits from the Pea Aphid, *Acyrthosiphon pisum*

**DOI:** 10.3390/ijms19082235

**Published:** 2018-07-31

**Authors:** Silvia G. del Villar, Andrew K. Jones

**Affiliations:** Department of Biological and Medical Sciences, Faculty of Health and Life Sciences, Oxford Brookes University, Oxford OX3 0BP, UK; s.garciadelvillar@gmail.com

**Keywords:** *Acyrthosiphon pisum*, alternative splicing, fipronil, GABA receptor, gene duplication, neonicotinoid

## Abstract

The insect GABA receptor, RDL (resistance to dieldrin), is a cys-loop ligand-gated ion channel (cysLGIC) that plays a central role in neuronal signaling, and is the target of several classes of insecticides. Many insects studied to date possess one *Rdl* gene; however, there is evidence of two *Rdls* in aphids. To characterise further this insecticide target from pests that cause millions of dollars’ worth of crop damage each year, we identified the complete cysLGIC gene superfamily of the pea aphid, *Acyrthosiphon pisum*, using BLAST analysis. This confirmed the presence of two *Rdl*-like genes (RDL1 and RDL2) that likely arose from a recent gene duplication. When expressed individually in *Xenopus laevis* oocytes, both subunits formed functional ion channels gated by GABA. Alternative splicing of RDL1 influenced the potency of GABA, and the potency of fipronil was different on the RDL1_bd_ splice variant and RDL2. Imidacloprid and clothianidin showed no antagonistic activity on RDL1, whilst 100 μM thiacloprid reduced the GABA responses of RDL1 and RDL2 to 55% and 62%, respectively. It was concluded that gene duplication of *Rdl* may have conferred increased tolerance to natural insecticides, and played a role in the evolution of insect cysLGICs.

## 1. Introduction

The insect γ-aminobutyric acid (GABA) receptor, known as RDL (resistant to dieldrin), plays a central role in neuronal signaling, and is involved in various processes, including regulation of sleep [1], aggression [2], and olfactory or visual learning [3,4]. The GABA receptor is a member of the cys-loop ligand-gated ion channel (cysLGIC) superfamily, which, in insects, also includes nicotinic acetylcholine receptors (nAChRs), histamine-gated chloride channels (HisCls), and glutamate-gated chloride channels (GluCls) [5]. CysLGICs consist of five subunits arranged around a central ion channel. Each subunit contains an N-terminal extracellular domain where neurotransmitter binding occurs (binding of GABA in the case of RDL), and four transmembrane (TM) domains, the second of which lines the ion channel [6].

RDL is also of interest as it is the target of several classes of highly effective insecticides such as cyclodienes (e.g., dieldrin), phenylpyrazoles (e.g., fipronil) and isoxazolines (e.g., fluralaner) [7]. In the genomic DNA of the model organism, *Drosophila melanogaster*, a mutation resulting in an alanine to serine substitution located in TM2 of *Rdl* was identified, which underlies resistance to several insecticides, including dieldrin, picrotoxin and fipronil [8,9]. This alanine to serine mutation, also found as alanine to glycine or to asparagine [10], has since been associated with insecticide resistance in various species, ranging from disease vectors (the malaria mosquito *Anopheles gambiae* [11,12]), to pests afflicting livestock (the horn fly *Haematobia irritans* [13]) or domesticated animals (the cat flea *Ctenocephalides felis* [14]) and crop pests (e.g., the planthopper *Laodelphax striatellus* [15]). Despite the emergence of insecticide resistance, RDL is still a potential target for insect control, since novel compounds have been developed that are unaffected by the TM2 resistance mutation [16].

Analyses of genome sequences have shown that insects of diverse species, such as *D. melanogaster*, *Musca domestica*, *Apis mellifera*, *Nasonia vitripennis*, and *Tribolium castaneum*, possess a single *Rdl* gene [5,17,18,19]. However, other insects, notably of the Lepidoptera order, possess more Rdl subunits. For example, *Chilo suppressalis* and *Plutella xylostella* have two *Rdl*-encoding genes [20,21], whilst *Bombyx mori* has three [22]. There is evidence that insects in other orders can also possess multiple *Rdl* genes. For instance, Southern blot analysis demonstrated the presence of two independent *Rdl* loci in the aphid, *Myzus persicae* [23]. In accordance with this, two candidate *Rdl* genes were observed in the genome of the pea aphid, *Acyrthosiphon pisum* [24]. Since many aphid species, such as *A. pisum*, are important crop pests which cause hundreds of millions of dollars’ worth of damage each year [24,25], it is prudent to study insecticide targets from these species in order to understand further mechanisms of resistance, as well as to facilitate the identification and development of improved insecticides that show specificity towards aphids whilst sparing non-target organisms.

We report here that the two *Rdl* genes in *A. pisum* encode for GABA-gated ion channels, upon which the insecticides fipronil and thiacloprid act as antagonists. We also show that *A. pisum* possesses an unusual cysLGIC gene superfamily, in that it lacks clear orthologues of LCCH3, GRD and CG8916. These subunits have been found in all other insect species so far where their complete cysLGIC superfamilies have been identified [5,19]. It was concluded that the duplicated *Rdl* in *A. pisum* may represent diversification, leading to the evolution of novel cysLGIC subunits in higher insects. 

## 2. Results

### 2.1. The A. pisum cysLGIC Superfamily Possesses Two Rdl Genes

**Figure 1:**
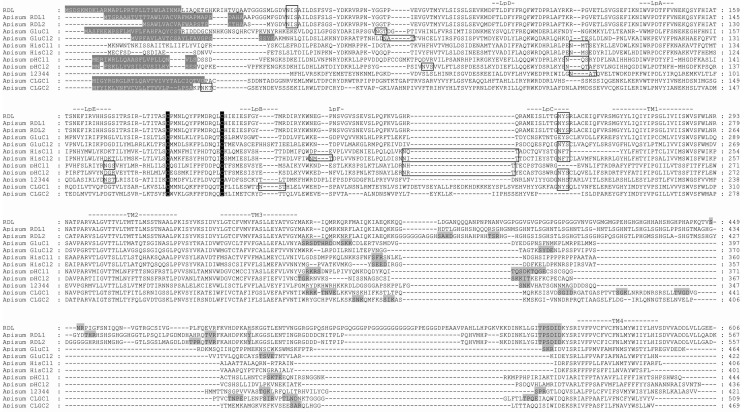
Protein sequence alignment of *A. pisum* cysLGIC subunits. *D. melanogaster* RDL_bd_ (RDL) is included for comparison. N-terminal signal leader peptides are shown in gray shading and white text. Loops implicated in ligand binding (LpA–F) are indicated, as well as the four transmembrane (TM) domains. The two cysteines forming the cys-loop are highlighted in black shading, and putative *N*-glycosylation sites are boxed. Potential cAMP, PKC, CK2 and tyrosine kinase phosphorylation sites are shown in gray shading. The sequences presented in this alignment can be found in the Supplementary Material.

Using tBLASTn, 22 candidate cysLGIC subunits were identified in the *A. pisum* genome. Eleven of these subunits are candidate nAChRs which have been previously described [24]; thus, in this report we focus on the remainder of the aphid cysLGIC superfamily. Alignment of their protein sequences (Figure 1) shows that the *A. pisum* subunits possess features common to members of the cysLGIC superfamily. These include: an extracellular N-terminal region containing distinct regions (loops A–F) [26] that form the ligand binding site; the dicysteine loop (cys-loop), which consists of two disulphide bond-forming cysteines separated by 13 amino acid residues; four transmembrane regions (TM1-4); and a highly variable intracellular loop between TM3 and TM4. As with other cys-loop LGIC subunits, the aphid sequences also possess potential *N*-glycosylation sites within the extracellular N-terminal domain, and phosphorylation sites within the TM3-TM4 intracellular loop.

**Figure 2:**
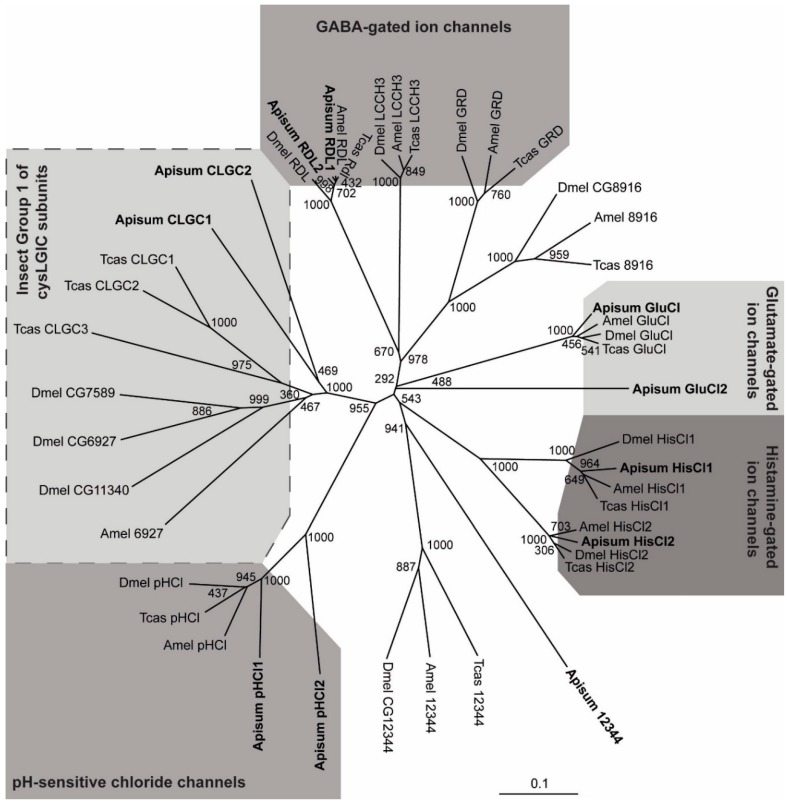
Tree showing relationships of *A. pisum*, *A. mellifera*, *D. melanogaster* and *T. castaneum* cysLGIC subunits. Numbers at each branch signify bootstrap values with 1000 replicates, and the scale bar represents substitutions per site. *A. pisum* cysLGICs are shown in boldface type.

A comparison of sequence identities between *A. pisum* and *T. castaneum* cysLGIC subunits (Table 1), as well as the use of a phylogenetic tree with *A. pisum*, *T. castaneum* and *A. mellifera* cysLGICs (Figure 2), indicates orthologous relationships between the aphid, beetle, and honeybee subunits. To facilitate comparisons between species, *Acyrthosiphon* subunits were named after their *Tribolium* counterparts. For example, the aphid orthologs of *Tribolium* HisCl1 and Tcas 12344 were designated Apisum HisCl1 and Apisum 12344, respectively. *A. pisum* possesses two putative subunits belonging to Insect Group I (Figure 2), which consists of *Drosophila* CG7589, CG6927 and CG11340 [18]. These two subunits were denoted Apisum CLGC1 and Apisum CLGC2, similar to the equivalent subunits in *T. castaneum* [18], since the orthologous relationships of both aphid subunits are uncertain.

**Figure 3:**
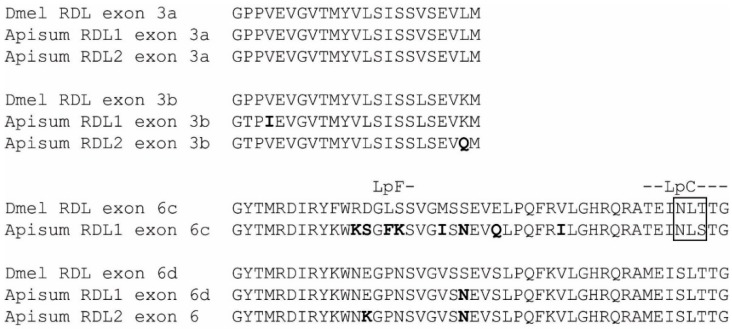
Splice variants of *A. pisum* and *D. melanogaster* RDL. Alternative splicing of exons 3 and 6. *Acyrthosiphon* residues that differ from those of the orthologous *Drosophila* exon are highlighted in bold. *N*-glycosylation sites are boxed and Loops C and F, which contribute to ligand binding, are indicated.

Two putative *Rdl* subunit genes were identified in the *A. pisum* genome, encoding for protein products denoted Apisum RDL1 and Apisum RDL2 (Figure 1 and Figure 2). Apisum RDL1 and Apisum RDL2 share notably high sequence identity with Tcas RDL, with 70% and 69%, respectively. However, Apisum RDL1 is considered the true ortholog of RDL in many other species, including *D. melanogaster*, *T. castaneum*, and *A. mellifera*, since it possesses alternative splicing at exons 3 (variants a or b) and 6 (variants c or d) [27], whereas Apisum RDL2 has alternative splicing only at exon 3 (Figure 3). Also, the NATPARVA peptide sequence preceding TM2 in RDL of many species is conserved in Apisum RDL1, whilst in Apisum RDL2 it is CATPARVS (Figure 1). The *A. pisum* genome also contains two subunits showing highest identity to the pH-sensitive subunit chloride channel [28], and thus, has been denoted Apisum pHCl1 and pHCl2 (Table 1). The identity of another subunit in the *A. pisum* genome was more difficult to assign, as it showed similar identity of 29% to Tcas GluCl and Tcas HisCl2. This subunit was tentatively denoted Apisum GluCl2, based on its slightly higher identity to Apisum GluCl1 as opposed to Apisum HisCl2 (Table 1), and that when considering the extracellular N-terminal region only, Apisum GluCl2 showed 33% identity to Tcas GluCl, as opposed to 30% identity to Tcas HisCl2. Interestingly, whilst *A. pisum* appears to have three duplicated subunits (Apisum RDL2, Apisum pHCl2 and Apisum GluCl2), the aphid lacks clear orthologs of LCCH3, GRD, and CG8916, which have been found in the genomes of other insects analysed to date (Figure 2) [5,17,18,19].

**Figure 4:**
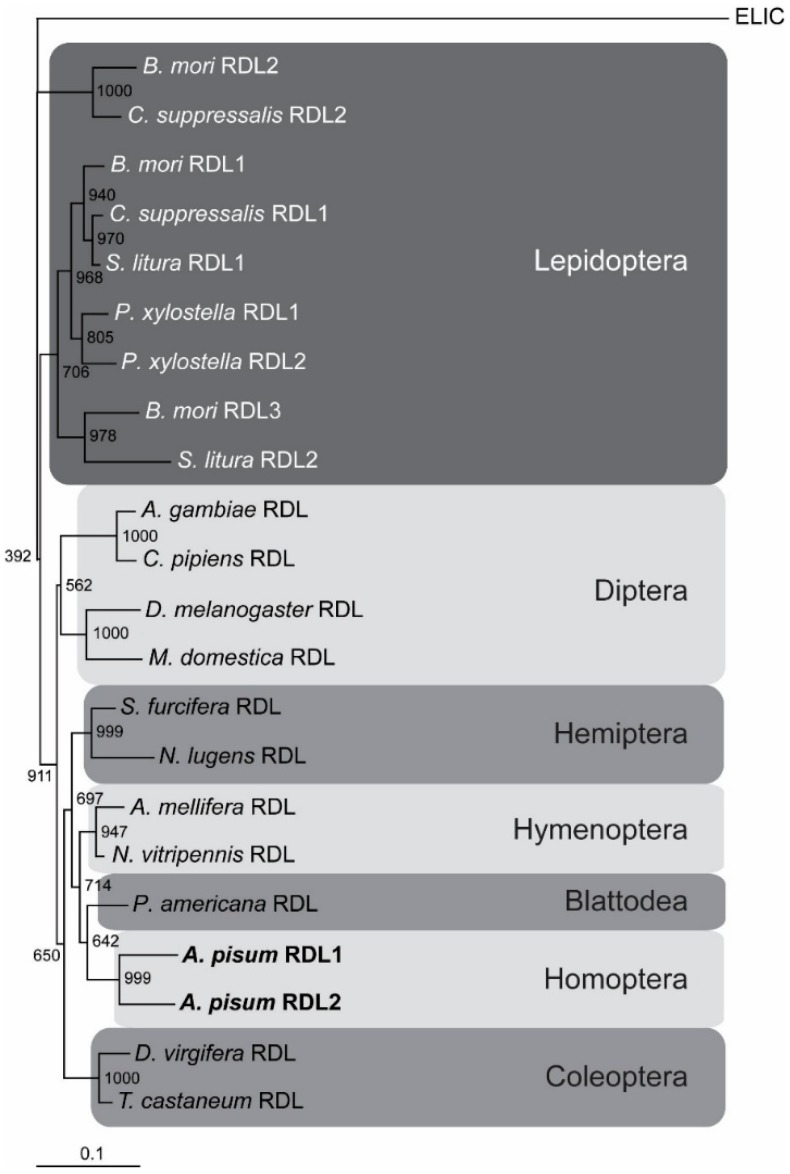
Tree showing relationships of RDL protein sequences from insects of various species. ELIC, which is an ancestral cysLGIC from the bacterium *Erwinia chrysanthemi* [29], was used as an outgroup. Numbers at each node signify bootstrap values with 1000 replicates, and the scale bar represents substitutions per site. *A. pisum* RDLs are shown in boldface type.

A phylogenetic tree was constructed using RDL peptide sequences from various insects (Figure 4). As previously observed [20], the RDLs segregated according to insect order, including the multiple RDL subunits found in Lepidoptera. When considering the RDL sequences of many species, both Apisum RDL subunits clustered close together. In line with this, the two aphid *Rdl* genes are arranged close together in the *A. pisum* genome, within 207 kb, indicating a recent duplication event.

### 2.2. Cloning and Functional Expression of Apisum Rdl1 and Apisum Rdl2

The full coding regions of Apisum *Rdl1* and Apisum *Rdl2* were amplified by reverse-transcriptase PCR, and cloned into the pCI plasmid. Ten clones for each subunit were sequenced. For Apisum *Rdl1*, one clone lacked exon 3, whilst for Apisum *Rdl2*, one clone lacking exon 3 and another missing both exons 2 and 3 were observed. *Rdl* variants lacking exon 3 were also observed in other insects such as *B. mori* [22]. Excision of the exons lead to a frame shift and the introduction of a premature stop codon, generating shortened open reading frames of 339 bp, 228 bp and 228 bp for Apisum *Rdl1*∆exon3, Apisum *Rdl2*∆exon3 and Apisum *Rdl2*∆exon2 + 3, respectively. The remaining nine clones of Apisum *Rdl1* were full length open reading frames consisting of 1704 bp encoding 567 amino acid residues. One of these clones encoded for the Apisum RDL1_ad_ splice variant, whilst the remaining eight were Apisum RDL_bd_, consistent with previous findings that bd is the predominant splice variant [30]. All the eight full length clones for Apisum *Rdl2* encoded for the exon3b variant; however, four of these clones had open reading frames of 1674 bp, whilst the other four had 1677 bp, encoding 557 and 558 amino acids, respectively. The difference in the open reading frame lengths is due to the presence of either a TVR or TEVR peptide motif in the TM3-TM4 intracellular domain, which were previously found in *A. mellifera* RDL, and were denoted variants 1 or 2, respectively [31]. No potential A-to-I RNA editing was observed in the twenty clones analysed.

**Figure 5:**
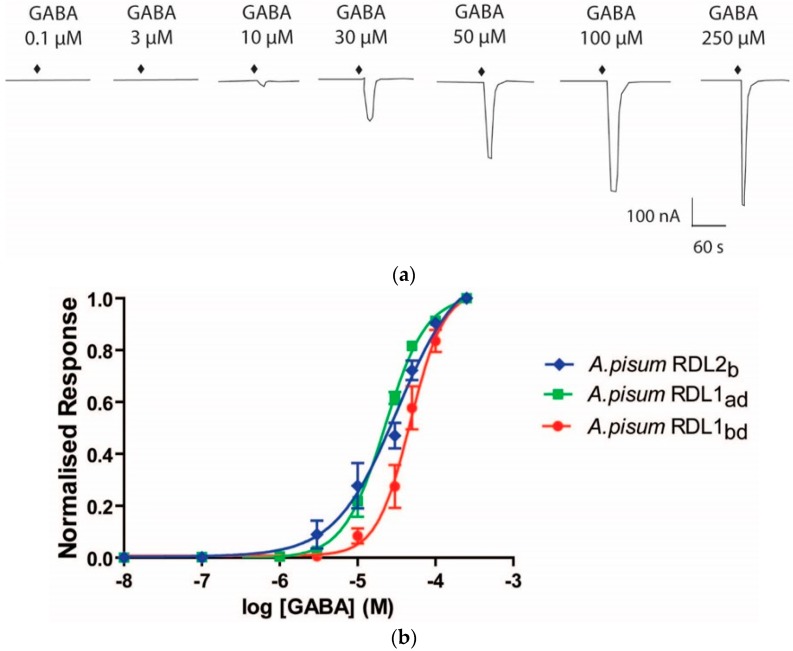
Responses to GABA in *X. laevis* oocytes expressing Apisum RDL. (**a**) Representative current trace of a GABA concentration response curve showing responses to GABA from 0.1–250 μM for Apisum RDL1_bd_; (**b**) GABA concentration response curves obtained for Apisum RDL1_ad_, Apisum RDL1_bd_, and Apisum RDL2_b_ variant 1. Data were normalised to the maximal response (250 μM). Data is the mean ± SEM from *n* = 4–5 oocytes from ≥ 3 different frogs.

*Xenopus laevis* oocytes were injected with plasmids encoding Apisum *Rdl1*_ad_, Apisum *Rdl1*_bd_ and Apisum *Rdl2*_b_variant1. Two-electrode voltage-clamp electrophysiology showed that oocytes injected with each of the *Rdl* constructs responded to GABA in a concentration-dependent manner (Figure 5a). GABA concentration curves were generated (Figure 5b) for each of the Apisum RDL constructs. The EC_50_ values of Apisum RDL1_ad_ and Apisum RDL1_bd_ were significantly different to each other (Table 2), and as is the case for *Drosophila* RDL [30], the bd splice variant for Apisum RDL1 has the highest EC_50_.

### 2.3. Antagonistic Actions of Fipronil and Neonicotinoids on Apisum RDL1 and Apisum RDL2

**Figure 6:**
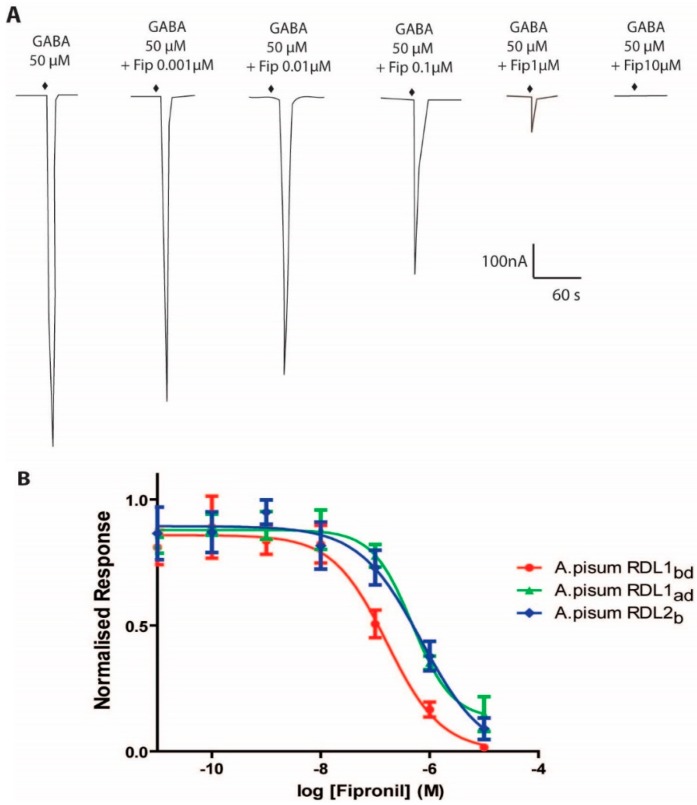
Effects of fipronil on currents activated by GABA at EC_50_ in *X. laevis* oocytes expressing either Apisum RDL1_ad_, Apisum RDL1_bd_ or Apisum RDL2_b_ variant 1. (**A**) Representative current traces showing the effect of 0.001–10 μM fipronil on the GABA response for Apisum RDL1_bd_. (**B**) Fipronil inhibition curves for Apisum RDL1_ad_, Apisum RDL1_bd_ or Apisum RDL2_b_ variant 1. Each data point was normalised to the maximum GABA response. Data are the mean ± SEM from *n* = 5 oocytes from ≥3 different frogs.

The actions of insecticides (fipronil and neonicotinoids) on Apisum RDL1 and Apisum RDL2 expressed in *Xenopus* oocytes were measured. Fipronil acted as an antagonist on both aphid RDLs, and inhibition curves were generated (Figure 6). The IC_50_ values for Apisum RDL1_bd_ and Apisum RDL2_b_ were significantly different from each other (Table 2).

**Figure 7:**
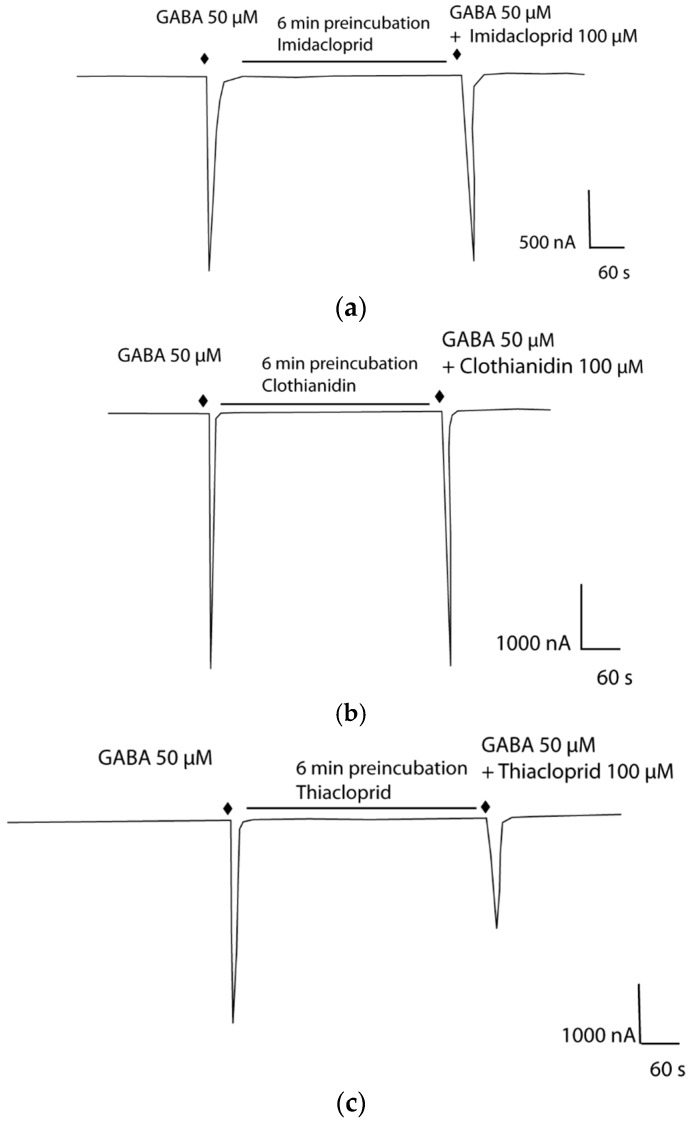
Representative current traces showing effects of 100 μM (**a**) imidacloprid, (**b**) clothianidin and (**c**) thiacloprid on GABA-induced currents (at EC_50_ concentration) in *X. laevis* expressing Apisum RDL1_bd_.

The neonicotinoid, imidacloprid, has been shown to act as an antagonist of heterologously expressed RDL [12]. We investigated to see whether imidacloprid also acted on the *A. pisum* RDLs. Unlike for *An. gambiae* and *A. mellifera* RDLs [12,31], imidacloprid at 100 μM had no detectable effect on responses of Apisum RDL1_ad_ or Apisum RDL_bd_ induced by GABA at EC_50_ concentration (Figure 7a) or at 1 mM. We therefore tested to see whether other neonicotinoids showed any actions on the aphid RDLs. Similar to imidacloprid, clothianidin also had no antagonistic actions on responses induced by GABA, either at EC_50_ concentration (Figure 7b) or at 1 mM. However, thiacloprid reduced the GABA-induced responses of Apisum RDL1_bd_ and Apisum RDL2_b_ var 1 to 55% and 62%, respectively (Figure 7c, Table 2).

## 3. Discussion

We report here the cloning and functional expression of two RDL subunits from the aphid, *A. pisum*, which is a significant pest of legume crops [25]. Phylogenetic analysis and their close proximity in the aphid genome suggest the two *Rdl* genes arose from a recent duplication event. Insects of the Lepidoptera order also have more than one *Rdl* gene. For example *P. xylostella* has two Rdl genes appearing to originate from a recent duplication [21,22]. In contrast, RDL1s of *B. mori* and *C. suppressalis* co-segregate, as is also the case for RDL2s of the same species [20], perhaps reflecting more distant gene duplications, with a second duplication event giving rise to RDL3 in *B. mori* [22].

*A. pisum* possesses the most unusual cysLGIC gene superfamily characterised to date, in that as well as having a duplicated *Rdl* gene, it also possesses duplicates of *pHCl* and *GluCl* subunits (Figure 2, Table 1). However, this does not result in an expanded cysLGIC gene superfamily, as no LCCH3, GRD or CG8916 subunits were detected in the *A. pisum* genome. This feature appears to be particular to the aphid, since insects of the Lepidoptera order have at least the LCCH3 and GRD subunits, as shown by *B. mori* [22]. With the cys-LGIC superfamily of *A. pisum* being the most evolutionary ancient characterised to date [32], it is tempting to speculate that duplication of *Rdl*, *pHCl* and *GluCl* represent diversification leading to the generation of *LCCH3*, *GRD*, and *CG8916* in more highly evolved insects. A similar finding was noted when characterising *A. pisum* nAChRs, where it was concluded that the α5 subunit was the newest member of the insect core group of nAChR subunits [24].

As with RDL of many other insect species, Apisum RDL1 has alternative splicing at exons 3 and 6, giving rise to four possible variants [10,27]. Functional expression of Apisum RDL variants showed that alternative splicing diversifies the functional properties of aphid RDL, as demonstrated by significantly different GABA EC_50_ values for Apisum RDL_ad_ and Apisum RDL_bd_. The use of differential splice sites can generate TM3-TM4 intracellular loops of varying length [10]. In the miridbug, *Cyrtorhinus lividipennis*, this can effectively create a 31 amino acid insertion, which decreased sensitivity to fipronil [33]. For the aphid RDL, only Apisum RDL2 was found to have variants where the intracellular loop varied in length. Here, an insertion of a single amino acid (TVR to TEVR) was identified. We did not functionally characterise these variants, as we have already shown that they have similar responses to GABA and fipronil in *A. mellifera* RDL [31]. With no potential A-to-I RNA editing isoforms detected, the extent of functional diversification of aphid RDL is less than that of other insects such as *D. melanogaster* and *An. gambiae*, which have at least 8 and 24 isoforms, respectively, arising from RNA editing [30,34]. RNA editing of *An. gambiae* RDL was found to influence the actions of ivermectin [34]. Without RNA editing, the aphid RDLs lack this mechanism to potentially alter target site sensitivity to insecticides. 

It has been previously noted that duplicated RDLs possess an amino acid substitution at the 2′-position of TM2, which is associated with insecticide resistance. For example, RDL2 of *C. suppressalis* possesses 2′ serine, instead of the highly conserved alanine present in RDL1 [20], an amino acid change found in dieldrin-resistant insects [8]. For *B. mori*, either alanine, serine, or glutamine are present at 2′ in RDL1, RDL2 and RDL3, respectively [22]. Consistent with previous findings in the aphid, *M. persicae* [23], we found that alanine (in RDL1) or serine (in RDL2) were present at 2′ in *A. pisum* RDLs. Functional expression of *C. suppressalis* RDLs showed that the alanine-to-serine substitution decreased sensitivity to dieldrin, but that both RDLs had similar IC_50_s in response to fipronil [20]. Studies of RDL from other insect species, such as *Nilaparvata lugens*, have also shown that alanine-to-serine mutation does not affect the antagonistic action of fipronil [35]. In contrast, we found that Apisum RDL1_bd_ and Apisum RDL2_b_ had significantly different fipronil IC_50_s. Apisum RDL2 has the amino acid sequence CATPARVS at TM2, which differs to NATPARVS present in *C. suppressalis* RDL2 [20]. Perhaps the unusual presence of the cysteine residue accounts for Apisum RDL2 showing lower sensitivity to fipronil. However, Apisum RDL1_ad_, which possesses NATPARVA at TM2, has a similar IC_50_ to Apisum RDL2_b_, suggesting that the cysteine residue does not underlie the differential sensitivity to fipronil. Apisum RDL1_ad_ and Apisum RDL1_bd_ differ by four amino acid residues located in the N-terminal extracellular domain, which is not associated with the actions of fipronil. Further experiments, such as site-directed mutagenesis, are required to clarify the basis of the differential sensitivity of Apisum RDL1_bd_ and Apisum RDL2_b_ to fipronil. It will be of interest to see whether differential expression of the aphid RDLs and their splice variants are associated with resistance to insecticides such as fipronil. It is also tempting to speculate that the evolution of insect cysLGICs may have been driven, in part, by gene duplication events conferring increased tolerance to naturally-found compounds with insecticidal properties. 

The neonicotinoid, imidacloprid, was shown to reduce GABA-induced responses in cultured honey bee Kenyon cells [36]. In line with this, more recent studies have shown that 100 μM imidacloprid acted as an antagonist of *An. gambiae* and *A. mellifera* RDL expressed in *Xenopus* oocytes [12,31]. We show here that imidacloprid at 100 μM has no antagonistic actions on Apisum RDL1 or Apisum RDL2, highlighting the fact that RDL can respond to neonicotinoids in a species-dependent manner. In addition, no reduction in GABA response was observed with clothianidin. We found, however, that thiacloprid was able to reduce GABA responses to a similar degree in Apisum RDL1_bd_ and Apisum RDL2_b_. Both imidacloprid and clothianidin are nitro-substituted neonicotinoids, whilst thiacloprid is cyano-substituted [37]. Perhaps this structural difference may underlie the differential actions of the neonicotinoids on the aphid RDLs. The concentration of thiacloprid required to antagonise Apisum RDLs is notably high, and it remains to be determined whether aphid RDL plays any role in the insecticidal effects of neonicotinoids.

In conclusion, two RDL subunits in the aphid *A. pisum*, which appear to be the result of a recent gene duplication event, were cloned and expressed in *X. laevis* oocytes. The heterologous expression of both aphid RDLs may provide a useful screening tool for the discovery of novel insecticidal compounds. This, in addition to screening against RDLs that have been cloned from other species such as *C. suppressalis* (a crop pest) [20], *C. lividipennis* (a predator of crop pests) [33] and *A. mellifera* (a pollinator) [31], may facilitate the identification of compounds which are selective for insect pests but benign for beneficial species. Furthermore, using expressed RDL with the 2′ mutation [15,35] in these screens can highlight novel compounds that are still active on insects with the TM2 mutation as an important step in managing resistance.

## 4. Materials and Methods

### 4.1. Isolation of Rdl1 and Rdl2 from A. pisum

The sequence of Apisum RDL1 identified from the *A. pisum* genome has been previously reported as a predicted gamma-aminobutyric acid receptor subunit beta isoform X1 (XP_001947125) [24]. A second potential RDL subunit was also reported (PREDICTED: similar to GABA receptor, XP_001947277); however, this sequence lacks the highly variable N-terminal signal leader peptide. In order to clone the full length of the second *Rdl* subunit, the tBLASTn program [38] was used to search sequence data of the aphid *Myzus persicae* available at AphidBase (Available online: https://bipaa.genouest.org/is/aphidbase/). This identified a *M. persicae* sequence (MYZPE13164_G006_v1.0_000138140.1_pep) with a signal peptide, which was then used to identify the equivalent N-terminus and signal peptide in the *A. pisum* genome using tBLASTn. The sequences of Apisum RDL1 and Apisum RDL2 have been submitted to NCBI (Available online: https://www.ncbi.nlm.nih.gov/), and have the accession numbers MH357526 and MH357527, respectively.

Total RNA was extracted from 12 adult *A. pisum* (taken from a lab colony and provided by Jim Goodchild at Syngenta) using Trizol (Fisher Scientific, Loughborough, UK) following the manufacturer’s protocol. First-strand cDNA was synthesized using the GoScript^TM^ Reverse Transcription System (Promega, Southampton, UK). The coding sequences of Apisum *Rdl1* and Apisum *Rdl2* were amplified from this cDNA by a nested PCR approach using the Q5^®^ High-Fidelity PCR Kit (New England Biolabs, Ipswich, MA, USA), where the first PCR reaction was used at a final dilution of 1 in 5000 as template for the second nested PCR reaction. For Apisum *Rdl1*, the first PCR reaction used the following primers: N-terminal 5′- CGCCGCCACGCCCGAGC-3′ and C-terminal 5′-GGCGCAAAGTCTGCGAATAAG-3′. The second reaction used: N-terminal 5′-GTCTAGAATGACCGGCCGCGCCGCG-3′ and C-terminal 5′ AGCGGCCGCCTACTTGTCCGCCTGGAGCA-3′. For Apisum *Rdl2*, the first PCR reaction used the following primers: N-terminal 5′-CGCCGGCACTCTTCTTCTTC-3′ and C-terminal 5′-TATGTAACACTGTAACCGATGAG-3′. The second reaction used: N-terminal 5′-GTCTAGAATGTCCGCGTGGCTGGTGG-3′ and C-terminal 5′-TGCGGCCGCTCAGTCCGCTCCCAGCAGTA-3′. Underlined sequences are *Xba*I and *Not*I, respectively, which were used to clone the aphid *Rdl* cloning sequences into the pCI vector (Promega). Apisum *Rdl* clones were sequenced at SourceBioscience (Available online: https://www.sourcebioscience.com/).

### 4.2. Sequence Analysis

The multiple protein sequence alignment was constructed with ClustalX [39] using the slow-accurate mode with a gap-opening penalty of 10 and a gap-extension penalty of 0.1, and applying the Gonnet 250 protein weight matrix. The protein alignments were viewed using GeneDoc (Available online: http://www.nrbsc.org/gfx/genedoc/index.html). Identity and similarity values were calculated using the GeneDoc program. Signal peptide cleavage sites were predicted using the SignalP 4.1 server [40], and membrane-spanning regions were identified using the TMpred program (Available online: http://www.ch.embnet.org/software/TMPRED_form.html). The PROSITE database [41] was used to identify potential phosphorylation sites. The phylogenetic trees were constructed using the neighbor-joining method and bootstrap resampling, available with the ClustalX program, and then displayed using the TreeView application [42].

### 4.3. Preparation and Expression of Apisum RDL1 and Apisum RDL2 in X. laevis Oocytes and Two-Electrode Voltage-Clamp Electrophysiology

Functional studies of Apisum RDL1_ad_, Apisum RDL1_bd_ and Apisum RDL2_b_ variant 1 were performed using the *X. laevis* expression system and two-electrode voltage-clamp electrophysiology. Stage V and VI *X. laevis* oocytes were harvested and rinsed with Ca^2+^ free solution (82 mM NaCl, 2 mM KCl, 2 mM MgCl_2_, 5 mM HEPES, pH 7.4), before defolliculating with 1 mg/mL type IA collagenase (Sigma, St. Louis, MO, USA) in Ca^2+^ free solution. Defolliculated oocytes were injected with 2.3 ng (23 nL) Apisum *Rdl* plasmid DNA into the nucleus of the oocyte and stored in standard Barth’s solution (supplemented with 50 µg/mL neomycin and 10 µg/mL penicillin/streptomycin) at 17.5 °C.

Oocytes 1–7 days post-injection were placed in a recording chamber and clamped at −60 mV with 3 M KCl filled borosilicate glass electrodes (resistance 0.5–5 MΩ) and an Oocyte Clamp OC-725C amplifier (Warner Instruments, CT, USA). Responses were recorded on a flatbed chart recorder (Kipp & Zonen BD-11E, Delft, The Netherlands). Oocytes were perfused with standard oocyte saline (SOS; 100 mM NaCl, 2 mM KCl, 1.8 mM CaCl_2_, 1 mM MgCl_2_, 5 mM HEPES, pH 7.6) at a flow rate of 10 mL/min. Oocytes were selected for experiments if stable after three or more consecutive challenges of GABA at EC_50_ concentration. The GABA EC_50_ concentration was determined using GABA concentration response curves, which were generated by challenging oocytes to increasing concentrations of GABA in SOS, with 3 min between challenges. Curves were calculated by normalising the GABA current responses to maximal responses induced by GABA before and after application.

Insecticides were initially diluted in dimethylsulphoxide (DMSO), before diluting to final concentrations in SOS. Final concentrations of 1% DMSO did not affect electrophysiological readings.

Fipronil inhibition curves were measured by pre-incubating the oocytes with fipronil in SOS for 3 min, immediately followed by a combination of fipronil and the respective EC_50_ GABA concentration (20 μM for Apisum RDL1_ad_, 50 μM for Apisum RDL1_bd_ and 30 μM for Apisum RDL2_b_ variant 1), until the maximum response was observed. This was followed by a wash step for 3 min in SOS and incubating the oocyte with 250 μM GABA, before repeating with increasing concentrations of fipronil. Inhibition curves were calculated by normalising the responses to the previous control response induced by 250 μM GABA.

For measuring the antagonistic actions of neonicotinoids, oocytes were initially incubated with a perfusion of the neonicotinoid in SOS for 6 min, before challenging with a combination of neonicotinoid and either the respective EC_50_ GABA concentration or 1 mM GABA.

### 4.4. Data Analysis

Data are presented as mean ± SEM of individual oocytes from three or more separate frogs. The concentration of GABA required to evoke 50% of the maximum response (EC_50_), the concentration of fipronil required to inhibit 50% of the maximal GABA response (IC_50_), and the Hill coefficient (nH) were determined by non-linear regression using Graphpad Prism 5 (Graphpad Software, CA, USA). Statistical significance was determined as *p* < 0.05, performed using one-way ANOVA (Graphpad Software, CA, USA).

## Figures and Tables

**Table 1:** Percentage identity/similarity between *A. pisum* and *T. castaneum* cysLGIC subunit protein sequences.

Subunit	Apisum RDL1	Apisum RDL2	Apisum GluCl1	Apisum GluCl2	Apisum HisCl1	Apisum HisCl2	Apisum pHCl1	Apisum pHCl2	Apisum CLGC1	Apisum CLGC2	Apisum 12344
Apisum RDL1	-	83/87	25/38	23/38	20/34	20/34	15/28	15/29	16/31	17/29	15/30
Tcas RDL	70/73	69/72	28/43	27/44	23/40	24/40	19/34	18/36	20/36	19/34	19/36
Apsium RDL2	83/87	-	25/37	23/38	20/35	20/34	16/28	15/29	17/31	17/28	15/31
Apisum GluCl1	25/38	25/38	-	29/44	26/42	25/42	20/38	19/38	23/37	18/36	17/34
Tcas GluCl	25/38	25/38	74/80	29/44	26/42	26/42	21/38	20/39	22/36	19/37	18/34
Apisum GluCl2	23/38	23/38	29/44	-	26/46	28/47	18/37	20/38	24/39	20/35	18/35
Apisum HisCl1	20/34	20/34	26/42	26/46	-	49/63	18/34	18/34	18/33	18/32	19/34
Tcas HisCl1	20/35	21/36	27/43	27/47	72/78	55/69	18/35	19/35	19/34	18/33	20/35
Apisum HisCl2	20/34	20/34	25/42	28/47	49/63	-	18/34	19/36	20/35	17/32	20/35
Tcas HisCl2	22/34	22/34	26/43	29/48	48/63	79/84	18/35	19/38	19/36	19/34	20/35
Apisum pHCl1	15/28	16/28	20/38	18/37	18/34	18/34	-	49/64	21/36	19/34	13/30
Tcas pHCl	16/30	17/31	22/40	20/39	19/36	19/37	66/74	51/66	21/35	19/33	15/32
Apisum pHCl2	15/29	15/29	19/38	20/38	18/34	19/36	49/64	-	20/37	19/34	15/32
Apisum CLGC1	16/31	17/31	23/37	24/39	18/33	20/35	21/36	20/37	-	36/53	15/30
Tcas CLGC1	18/31	18/31	20/37	21/37	17/33	19/36	18/31	19/33	35/49	27/44	13/29
Apisum CLGC2	17/29	17/28	18/36	20/35	18/32	17/32	19/34	19/34	36/53	-	13/31
Tcas CLGC2	16/29	17/30	19/35	21/37	16/32	17/33	16/30	17/31	31/48	25/43	13/28
Apisum 12344	15/30	15/31	17/34	18/35	19/34	20/35	13/30	15/32	15/30	13/31	-
Tcas 12344	19/33	20/34	21/38	23/41	23/42	26/46	18/34	20/37	19/37	16/32	20/37
Tcas CLGC3	16/30	16/30	19/36	20/38	18/36	19/38	19/35	19/37	29/49	22/44	13/31
Tcas GRD	27/41	27/41	24/40	22/38	21/36	21/38	16/31	18/33	19/34	18/31	13/31
Tcas LCCH3	28/44	28/43	26/44	27/44	23/38	25/41	16/32	16/32	19/37	20/37	17/33
Tcas 8916	24/40	24/39	23/38	22/36	19/33	21/35	15/30	15/31	19/33	17/32	14/27

Proposed orthologues in *A. pisum* and *T. castaneum* are underlined.

**Table 2:** Effects of GABA on membrane currents from *X. laevis* oocytes expressing *A. pisum* RDL, with maximum amplitude (*I*_max_), EC_50_ and hill coefficient (nH) displayed. The *I*_max_ was obtained from the initial 250 μM GABA response measured from eggs clamped at −60 mV. Also shown are the effects of fipronil and the neonicotinoids imidacloprid (IMI), clothianidin (CLO), and thiacloprid (THI) on GABA EC_50_ induced membrane currents. IC_50_ values are shown for fipronil, as well as the fraction of response to GABA at EC_50_ after exposure to 100 μM neonicotinoid. All data are the mean ± SEM of 4-5 oocytes from ≥3 different frogs. [–] indicates that this value was not measured.

Subunit	*I*_max_ (μA)	GABA		Fipronil IC_50_ (μM)	% of GABA Response with 100 μM:		
		EC_50_ (μM)	nH		IMI	CLO	THI
Apisum RDL1_ad_	1.6 ± 0.6	23 ± 1.7 ^1^	1.6 ± 0.1	0.64 ± 0.15	100	100	–
Apisum RDL1_bd_	1.4 ± 1.3	51 ± 9.2 ^1^	2.0 ± 0.3	0.18 ± 0.06 ^2^	100	100	55 ± 7
Apisum RDL2_b_ var 1	1.4 ± 1.5	28 ± 5.4	1.0 ± 0.1	0.72 ± 0.19 ^2^	–	–	62 ± 2

^1, 2^ These GABA EC_50_ or fipronil IC_50_ values are significantly different (*p* < 0.05) using one-way ANOVA with Bonferroni’s multiple comparison test.

## Supplementary Materials

Supplementary materials can be found at http://www.mdpi.com/1422-0067/19/8/2235/s1.

## Abbreviations

cysLGIC	cys-loop ligand-gated ion channel
RDL	resistant to dieldrin
nAChR	nicotinic acetylcholine receptor
