# Supplementary material for: Cloning and Functional Characterisation of the Duplicated RDL Subunits from the Pea Aphid, Acyrthosiphon pisum
Source: Int J Mol Sci. 2018 Jul 31;19(8):2235. doi: 10.3390/ijms19082235 (PMC6121307; doi:10.3390/ijms19082235)
Supplement: Supplementary file 1 [file ijms-19-02235-s001.pdf]

Acyrthosiphon pisum cysLGIC peptide sequences

>Apisum_RDL1
MTGRAAHTVTTTAWLVACVAFMAPMAPPDAHAAAPGGGMFGDVNISAILDSFSLSYDKRVRPNYGGTP
IEVGVTMYVLSISSLSEVKMDFTLDFYFRQFWTDPRLAFTKRPGVETLSVGSEFIKNIWVPDTFFVNE
KQSYFHIATTSNEFIRIHHSGSITRSIRLTITASCPMNLQYFPMDRQLCHIEIESFGYTMRDIRYKWN
EGPNSVGVSNEVSLPQFKVLGHRQRAMEISLTTGNYSRLACEIQFVRSMGYYLIQIYIPSGLIVIISW
VSFWLNRNATPARVALGVTTVLTMTTLMSSTNAALPKISYVKSIDVYLGTCFVMVFASLLEYATVGYM
AKRIQMRKNRFLAIQKIAEQKKGGHDTLGHGHSHQQQRSGHGNMSGHNTLSGHNTLSGHNTLSGHNTL
SGHNTLSHGLSGHGLPPSHGTMAGHGGYDTHRHSHSGHGGHGGTLPSQILPGDMDHAHRQTVRFKAHD
PKNYLKGGSLENTINGRGDDDCNITPLPPQHVMHPSKDINKLYGITPSDIDKYSRIVFPVCFVCFNLM
YWIIYLHISDVVADDLVLLQADK
>Apisum_RDL2
MSAWLVACVAFWAPSTPCTHAASMYGDVNISAILDSFSGSYDKRVRPNYGGTPVEVGVTMYVLSISSL
SEVQMDFTLDFYFRQFWTDPRLAFTKRPGVETLSVGSEFIKNIWVPDTFFVNEKQSYFHIATTSNEFI
RIHHSGSITRSIRLTITASCPMNLQYFPMDRQLCHIEIESFGYTMRDIRYKWNKGPNSVGVSNEVSLP
QFKVLGHRQRAMEISLTTGNYSRLACEIQFVRSMGYYLIQIYIPSGLIVIISWVSFWLNRCATPARVS
LGVTTVLTMTTLMSSTNAALPKISYVKSIDVYLGTCFVMVFASLLEYATVGYMAKRIQMRKNRFLAIQ
KIAEQKKGGGGGGGAGGHSAHDGHSHAHPHHTSRHGHGHGSISGHSTLSGHGTLSGGHGPQSGHGTLP
HGMSSHGLASHGTMSSHGYDGGGGGHRHSHMGHGGTLSGSLMAGDLDHTPRQTVRFKAHDPKNYLKGG
SLENTINGRGDDDNITPIPTQHVMHPNKDINKLYGITPSDIDKYSRIVFPVCFICFNLMYWIIYLHIS
DVVADDLVLLGAD
>Apisum_GluCl
MASFKEWPSFLMVFLFAHFAQYCSTRIDDDGCNNHKGNSQHRCVPKVNYRHKEKEVLDQILGPGSYDA
RIRPSGVNGTDGPTIVHINLFVRSIMTISDNKMEYSVQLTFREQWMDERLKFNDYNGKMKYLTLTEAN
RVWMPDLFFSNEKEGHFHNIIMPNVYIRIFPNGLVLYSIRISLTLSCPMNLKLYPLDRQTCSLRMVSG
WTTDDLVFLWKEGDPVQVVKNLHLPRFTLEKFLTDYCNSKTNTGEYSCLKVDLLFKREFSYYLIQIYI
PCCMLVIVSWVSFWLDQSAVPARVSLGVTTLLTMATQTSGINASLPPVSYTKAIDVWTGVCLTFVFGA
LLEFALVNYASRSDTHRDDMSKKCDLERTVSMDVGDYEDGPNSFNMKPLMRPELMMSRDKMSQIIHQT
PPMEKNSCWKSWMSKFPTRSKRIDVISRILFPMVFAMFNMSYWSTYLFREGVDEDK
>Apisum_GluCl2
MVPFAVLAVTCSAYLFAVGPGSSYGAKMNHLQILDSLLKKNYDRRATPTNHLDNATTVHCELYIRSFG
SISPVTMDYEVDLYLRQKWQDARLQHKDITESLDLNDPNLVKAIWKPEVYFPNAKHAEFQFVTVPNVL
IRIKPDGDILYMLRRLKLTFSCMMDLSKFPLDNQICTMEVASCEFHHSKTIEELRLEWKNTDPVLMAK
GLRMPQFEIVDIVPSDCQESFQIGNYSCLVAQFYLSRSVGFHLVQSYLPTMLIVVISWVSFWMDVDSV
PGRTTLGVTTLLAVGSQSSGIQSGLPQVSYVKAIDVWMGTCTAFVFCALLEFTVVNYMWRKLRPDILR
NILTEPVNGGTAGTKSYDENLPSSPVIPVSVVITVLQQECAYSTSVETNTVLARKIDEYSRLLFPVAF
IAFNALYWPYYLHN
>Apisum_HisCl1
MKNWNTNKISSAIITKLIIFLYVIQFRYSKCLEFRDILPEDAKQYDKMRPPKIDGTATKVHFHVTVMG
LDSIDENAMTYAADIFFAQTWKDHRLRLPENMTSEYRLLEVDWLKNIWRPDSFFKNAKAVTFQTMTIP
NHYMWLYKDKTILYMVKLTLTLSCAMNFMIYPHDTQECKLQMESVSHTTDDMIFQWDPEVPLVVDENI
ELPQLQLVKNQTADCTQVYSTGNFTYYLFHTYIPTCLIVIMSWVSFWIKPEAAPARVTLGVTSLLTLS
TQHAKSQAALPPVSYLKAVDTFMSACTVFVFMALMEYCLVNIVLGDSDMPPKQLNKKSFNFSFRSNLK
LNDGHSIPPPPRPPPAATAAALAATTALAQRNRTRAINIDRFSRVFFPLLFAVLNMTYWVIFARFL
>Apisum_HisCl2
MECFSDQSDIAESDNVVFETSLSLTDILPINPKNYDKNRAPKVQGQPTIVYFHVTVLSLDSINEESMT
YVADIFLAQSWRDQRLRLPENMSEDYRILDVEWLHDIWRPDCFFKNAKKVTFHEMSIPNHYLWLYHDK
TLLYMSKLTLVLSCAMKFESYPHDTQICSMMIESLSHTTHDLVFIWNMTDPLVVNPDIELPQLDISNN
ITTDCTIEYSTGNFTCLAVMFNLRRRLGYHLFHTYIPSALIVVMSWISFWIKPEAIPARVTLGVTSLL
TLATQNTQSQQSLPPVSYVKAIDIWMSSCSIFVFLSLMEFAVVNNYMGPVATKVMKGYSEEDIRGGDD
FKELDPKNRSPIRSVATVAAAPQYPTFCNGRAIALYIDKFSRFFFPFSFFILNVAYWTSFL

```
>Apisum_pHCl1
MERIWRLLQAASFLVPLLQNVLSDSSDVFENGKSDKEILDNLVNPNRYDKRLLPPVDDQDFCCGMKTP
QDVRVILPSANRHPIRKGKYEVEFLLQQQWYDPRLRYSNQSRYDFLNAIHHHGDIWVPDTYFIMHGDF
KASLIPVHFSLRIYRNGSVHYLMRRHLILSCQGILNIFPFDDPQCTFSMESVSYEMSAIKYVWKNDES
TLKKSPSLKTLNAYLDRNHTTECPSTGSWRGEVNLIFTRDRAFYFTTVFIPGIILVTSSFITFFLEWN
AVPARVMIGVTTMLNFFTTSNGFRSTLPVVSNLTAMNVWDGVCMCCIYASLLEFVLVNYVGRKRSDWP
LPIVYQNKDQDYKQITQSDKTQGECSSCGQGACTHSLLHNTCPSKTEEQNIRSNGGNKRKMPPHPIRI
AKTIDVIARITFPTAYAVFLIFFFIHYTSHPSHWSQ
>Apisum_pHCl2
MNFSKLLYVSILCVSILKYSESVQPKEVPFKFENGKSDKHILDHLLNTDIYDKRLLPPSYGPSIVNVS
VVLFTLSSPDESSLKYDIEFLLQQEWYDSRLKYPNQTAFSVLNGIHHQGDIWLPDTNFHMHGDFKPSQ
RPIRFTLNVYNDGRVEYTTRRHLMLVCEGSMKIFPFDQPLCTFSMESISYEKSDLKYVWKEDNFVLIK
SPNLKTSNAYLERNRTYECASTGSWRGNYSCLQVELIFSRDRTFYFATIFIPGLILVTSSFITFFLEW
DAVPARTIIGVTTMLNYFTTSNSFRRTLPDVANLTAMNVWDGVCMCFVFASFIEFVIVNCTGRRNVKS
NISSNAGSCSISKKITKEFKCPECAQNVCSHSLLIDVLPKVNEKIATKPSDQVHLAMRIDTVARLTFP
SAFVMFLAFFFFYYSANANNRAISVNTN
>Apisum_12344
MFHLVRSKDFYEMETEYNKNMLPGKDVDVSLSLHINRISGVDESKEEIALDVFLHVYWEDPRIWILGN
ATQVELTWDKDPKFWIPDLYIRQLREMKVLTLFQDLASIRLYDNSTLRISIGATIIIKCDMDFVLYPL
DVQTCAVDFGSCKYTLTDMKFHWRDDPLIFPSDFGDGFRLPRYVVSFVTDNRLNVIYYGDGNHSTARL
VITLSRELRSHLLESYLPSTLFVIMSWGSFVVMPEVVPGRMVLLVTTLLSLVTMFDTIRNNSPSALEL
KCIEVWLISCTLFVFLALVEYFIVLFGMRYDKHWRHKKRDLDGSGGAPSKPPLFGSNKVHATSGNNMA
GDDDSQQHMMTTNSGPVVVASTGKLRFQLITRHVILYCGSPRGTLDQVSLALFPLCFVIFSVMYWISY
LNESRLKALASVV
>Apisum_CLGC1
MIGYRGRGLRTLQAVTVIVYTALCIQTVQPACSDDNTADGGNRVEMLMWLTDPCRYDRWTRPIPPDGN
TKTGAPTRVSARVYVYFLGSVEAQQLHFTAHLLIRYRWRDDRLVHSSSKSSIQGENKLKERVWTPHVY
IVNEHDSNIMGSGRQDILVTVQPDGTVLYSARLKVSLLCMMNLQKFPFDQQTCPLILESWTYNSTHLE
LVWEHDSPAVLNSNLRMTEYNLVSIWTDETVSEYALLPSEDKHDKKKEYSPLSVEHVHYYGKFAGNYS
QLIVNFELEREVGHYIMDYYVPSIMLVVVSWVSFWLDPNAVPGRTTLGTSTMLTFITLSRNIGSSLPK
VSYIKATEIWFIVCTAFIFGSLVEFAFVNTIWRRKTNVELKKVNSKHIFKSTLTPQMQRKSISVDSGI
DRGATGASPTVTSGKLNRRDNRSRSLLTVGDVGPTVCTNPEPLENFSIRVPTLNDNKTGGGGGLFTLT
PQEIAQWIDQRSRIAFPAAFLVFNCFYWLYVYV
>Apisum_CLGC2
MFYIKLYNFVCVLLFTVVLPLPSAKPNKTCGSEYNDEVSSSEKILRWLTDPCRYDKTVAPGKLVAHNP
IVVFTRIHVYHLTSVYPRNLDFKVRILLQFRWKDVRLAFDNLAPMDSSEVVCQKGILERIWLPNVYIA
NEKHSLTVADMTEDLMVTVLPDGTVMLSVRLKTSVFCWVKLGRFPFDRQQCHLIMETCRYDTTQLVLE
WEVESPVTAALNDNSWVPYLTEYKLLKIIPHSSEHYVEPISLDPAREYGFYIMDYYVPGILLVTISWV
SFWMAPDATPARVAIGTSTMLTYFQLGVDTGSKLPNVSYIRSNDLWFIVCTAFIFLSLAEFAFVNTIW
RYGCHPVKLKSKSNKQMFKSSIKASKMSKGIVNNDRPRQTIAAGSLFLDGIRLQNNGTSELNVELPYT
MEMKAMGKVKFKVSEESARQLHGGLTTTAQQISIWIDEKSRIVFPASFILFNAVYWSLLWV
```
